# Canine leishmaniosis on Favignana Island: a prospective observational study on infection dynamics, clinical outcomes and field safety in dogs receiving both vaccination (LETIFEND^®^) and an antifeeding collar (SCALIBOR^®^)

**DOI:** 10.1186/s13071-026-07452-8

**Published:** 2026-05-30

**Authors:** Valentina Foglia Manzillo, Gaetano Oliva, Germano Castelli, Federica Bruno, Roberto Rosenthal, Serena Montagnaro, Ines Balestrino, Claudia La Rocca, Liliana Colombo, Manuela Gizzarelli

**Affiliations:** 1 Department of Veterinary Medicine and Animal Production, “Federico II” University, Naples, Italy; 2 C.Re.Na.L - National Reference Center for Leishmaniasis, Istituto Zooprofilattico Sperimentale Della Sicilia Palermo, Palermo, Italy; 3 DVM, Favignana, Italy; 4 MSD Animal Health Italy, Milan, Italy

**Keywords:** Canine leishmaniosis, LETIFEND^®^ vaccine, SCALIBOR^®^ collar

## Abstract

**Background:**

Canine leishmaniosis (CanL) is endemic in Italy and is an important veterinary and public health concern. Integrated preventive strategies combining vaccination and vector control are recommended in endemic areas, and field data supporting the safety and effectiveness of this concurrent use are limited.

**Methods:**

A longitudinal field study conducted on Favignana Island (Sicily, Italy) evaluated the concurrent use of a vaccine (LETIFEND^®^, Leti) and sandfly repellent collar (SCALIBOR^®^, MSD Animal Health). Client-owned dogs (154) were prescreened by clinical examination, serology, quantitative polymerase chain reaction (qPCR) and serological testing (SNAP^®^4Dx^®^ Plus, IDEXX). *Leishmania*-negative dogs (62) were enrolled in the study, vaccinated, fitted with a deltamethrin-impregnated sandfly repellent collar, and monitored every 6 months for 24 months (2 transmission seasons). Follow-up evaluations included clinical examination, serological testing (SNAP^®^ Leish^®^ Test, IDEXX), and conjunctival swab qPCR; sera were stored for subsequent indirect immunofluorescence antibody testing (IFAT). After 12 months, dogs that remained negative received a booster vaccination and replacement collar.

**Results:**

The proportion of dogs positive for CanL in the prescreen was 12.3% (19/154). Two dogs in the enrolled group (3.22%, 2/62) developed clinical signs compatible with CanL and tested positive (SNAP^®^Leish^®^,IDEXX) during the study. IFAT testing of enrolled dogs found low antibody titres in nine additional clinically healthy dogs corresponding to a cumulative infection incidence of 14.5% and an incidence density of 0.61% per dog-month. No local or systemic adverse reactions associated with the combined preventive protocol were recorded.

**Conclusions:**

Concurrent vaccination (LETIFEND^®^, Leti) and preventive collar administration (SCALIBOR^®^, MSD Animal Health) resulted in no adverse events, although there was a low incidence of clinical CanL and seroconversion over two consecutive transmission seasons. These findings support the use of integrated preventive strategies to limit the development of clinical disease in dogs living in endemic areas.

**Graphical Abstract:**

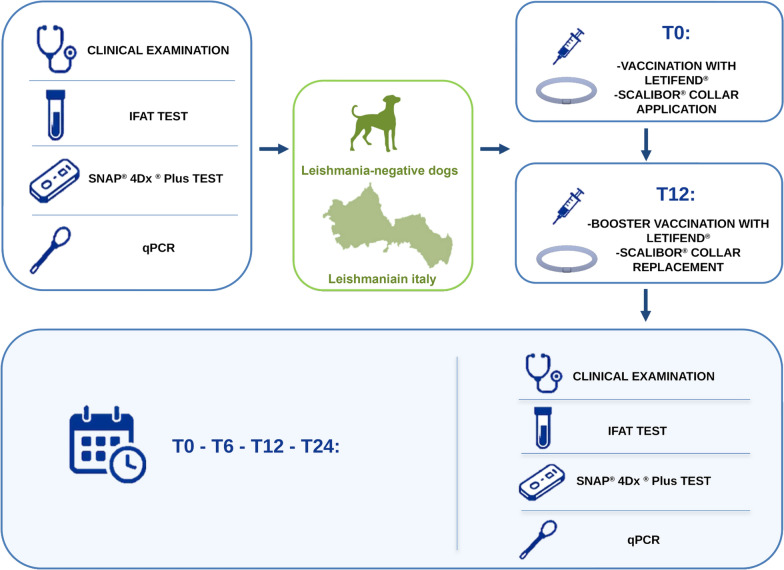

## Background

Leishmaniosis is classified as a neglected infectious disease by the World Health Organization (WHO) on the basis of its wide distribution and public health impact. The disease is caused by more than 20 species of protozoan parasites in the genus *Leishmania* (Kinetoplastida: Trypanosomatidae) and is transmitted by several species of phlebotomine sand flies [1].

The main clinical forms in people are: cutaneous leishmaniasis (CL), mucocutaneous leishmaniasis, and visceral leishmaniasis (VL), with differing severity, transmission dynamics, and geographical distribution. Visceral leishmaniasis is the most severe form and is fatal in up to 95% of untreated cases, with approximately 11,000 new autochthonous cases reported annually in endemic countries [1].

The incidence of VL is influenced by multiple factors, including warmer weather, vector and reservoir host population growth, increased contact between humans and animals, and human migration. Increasing pediatric VL cases are reported in Italy [2–4]. A multicentric retrospective study involving all Infectious Diseases Units (*n* = 12) in Tuscany region found an increase in human leishmaniasis incidence in cases per 100,000 inhabitants from 0.22 cases in 2018 to 1.81 in 2023. These results exceeded estimates derived from mandatory surveillance and hospitalization records between 2011 and 2016 and confirmed the increasing disease risk [5].

Canine leishmaniosis (CanL), caused by *Leishmania infantum*, is endemic in Italy, with an estimated median canine infection prevalence of 17.7% [6, 7], although it is > 30% in southern regions and in the islands of Sicily and Sardinia [6, 8]. CanL is clinically variable, ranging from asymptomatic infection to severe systemic disease with signs including anemia, thrombocytopenia, hyperproteinemia, renal and hepatic impairment, weight loss, hepatosplenomegaly, lymphadenopathy, and ocular and cutaneous lesions [9].

Dogs are at the center of CanL epidemiology because of the number of dogs and their interactions with people, and infection control in dogs is a public health priority. CanL control strategies require accurate diagnosis and treatment of infected animals combined with preventive measures that limit transmission. These measures include: sandfly population control, individual dog protection with topical insecticides and repellents, and dog vaccination [10].

There is only one licensed vaccine (LETIFEND^®^, Leti) for dogs against *L. infantum* in Italy, and this vaccine contains recombinant protein Q, obtained by genetic fusion of five antigenic determinants derived from four *L. infantum* MON-1 proteins. Vaccination efficacy was evaluated in a multicenter field trial involving 549 healthy seronegative dogs naturally exposed to *L. infantum* over two consecutive transmission seasons in endemic areas of France and Spain. Dogs were vaccinated at baseline and revaccinated 1 year later. Vaccinated dogs had a significantly lower incidence of clinical leishmaniosis incidence, with 72% efficacy. The risk of clinical disease in vaccinated dogs was reduced 5-fold, parasite detection was reduced 3.5-fold, and clinical signs occurrence was reduced 9.8-fold compared with unvaccinated dogs [11].

There are several approved options for sandfly repellency or antifeeding effect in Italy. Deltamethrin (SCALIBOR^®^, MSD Animal Health) collars provide long-lasting antifeeding efficacy against sand flies (up to 94%) for up to 364 days [12], with an associated insecticidal activity [13, 14]. Studies in Europe and Brazil have shown the potential for deltamethrin-impregnated collars to reduce *L. infantum* transmission in communities [15–17].

**Fig. 1:**
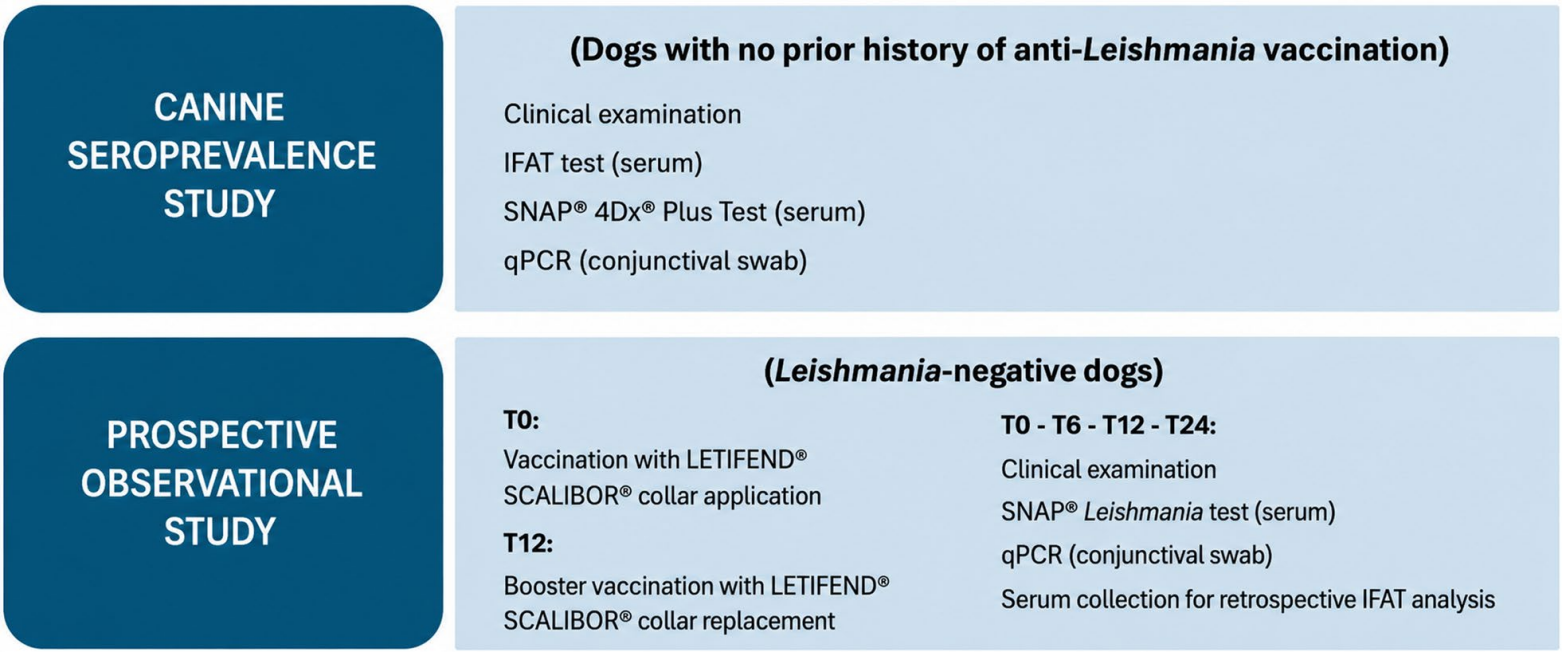
Experimental study design. The study comprised two sequential phases. (i) Canine seroprevalence study: dogs with no prior history of anti-Leishmania vaccination underwent clinical examination and were tested by IFAT on serum, SNAP^®^ 4Dx^®^ Plus on serum, and qPCR on conjunctival swab. (ii) Prospective observational study: Leishmania-negative dogs were enrolled and received LETIFEND^®^ vaccination and SCALIBOR^®^ collar at baseline (T0), with booster vaccination and collar replacement at T12. Follow-up visits were conducted at T0, T6, T12, and T24 and included clinical examination, SNAP^®^ Leishmania test (serum), qPCR on conjunctival swab, and serum collection for retrospective IFAT analysis

Canine leishmaniosis reduction recommends concurrent use of vaccination and antifeeding collars; however, additional data are needed on the impact of this approach under field conditions. Favignana Island provides an opportunity to investigate this concurrent use because of the local population of naturally exposed dogs living in a geographically confined endemic settings [18]. This study was designed as a prospective, single-arm observational cohort study on Favignana Island (Fig. 1) with the objectives of: (i) determining the CanL seroprevalence among owned dogs; (ii) to record the infection onset rate and clinical signs in *Leishmania*-negative dogs receiving concurrent LETIFEND^®^ vaccination and SCALIBOR^®^ collar administration followed by natural exposure conditions over a 2-year period; and (iii) to evaluate the safety of the concurrent use of LETIFEND^®^ vaccine and SCALIBOR^®^ collar in field conditions.

## Methods

### Study site

Favignana is the largest island (19.8 km^2^) of the Egadi archipelago, located 7 km off the western coast of Sicily in the Mediterranean Sea (37° 55′ 34″ N 12° 19′ 16″ E). The climate is temperate Mediterranean, with mild, rainy winters and hot, dry summers, with temperature peaks up to 31 °C (https://www.ilmeteo.it/portale/archivio-meteo/Favignana). Favignana has a permanent human population of around 5000 residents, mostly concentrated in the main town on the northern coast. Cats are the most abundant domestic species on the island, many living in semiferal colonies. There are about 1000 dogs, and goats and sheep, primarily in rural areas. There are no data regarding leishmaniasis prevalence on Favignana; however, surveys on other Mediterranean islands (Aeolian and Pelagie Islands) found significant prevalence of *Leishmania infantum* in both dogs and cats, highlighting the cats population as a potential secondary reservoirs [19, 20]. Canine seroprevalence > 30% and feline seroprevalence rates of 10–12% are reported in endemic areas in Sicily [7, 21]. Furthermore, wildlife in Sicily can harbor *L. infantum*, suggesting that sylvatic reservoirs contribute to the transmission cycle [22].

### Experimental study design and animals

#### Canine seroprevalence study

Owned dogs (154) with no history of anti-*Leishmania* vaccination were clinically examined, and a blood sample for IFAT and conjunctival swab for quantitative polymerase chain reaction (qPCR) were taken. In addition, all dogs were screened for co-infections (SNAP^®^ 4Dx^®^ Plus Test, IDEXX Laboratories, Westbrook, USA) including *Dirofilaria immitis* antigen and antibodies against *Borrelia burgdorferi*, *Ehrlichia canis/E. ewingii*, and *Anaplasma phagocytophilum/A.platys.*

#### Feline leishmaniosis

Stray cats (82) of both sexes were blood tested for *Leishmania spp* infection using IFAT.

#### Prospective observational study

*Leishmania*-negative dogs (62) found in the initial screening test were enrolled and completed the study. These dogs were all vaccinated (LETIFEND^®^) and a collar (SCALIBOR^®^) was applied according to product labels. All dogs were retested before vaccination, as per vaccine protocol, using the SNAP^®^ *Leishmania* test (IDEXX Laboratories, Westbrook, USA) to confirm test-negative status.

Dogs were monitored every 6 months for 2 years, and at each follow-up point, they were clinically examined and had a blood sample taken for *Leishmania* testing (SNAP^®^). In addition, serum was collected and frozen at −20 °C for subsequent *Leishmania* IFAT testing, and conjunctival swabs were taken for specific qPCR. Dogs exhibiting clinical signs possibly consistent with CanL or testing positive on either the *Leishmania* test and/or conjunctival qPCR, were removed from the study and specific anti-*Leishmania* treatment was recommended. At 1 year, all test-negative dogs received a booster vaccination and were administered a new collar.

### CanL and FeL parasitological diagnosis

Serological tests using IFAT assays were conducted according to the World Organisation for Animal Health (WOAH) Manual of Diagnostic Tests and Vaccines for Terrestrial Animals, CAP. 3.1.11 [23]. Sera were prepared by two-fold serial dilutions in phosphate-buffered saline (PBS) at pH 7.2 and placed in antigen-coated wells. *L. infantum* promastigotes (MHOM/IT/80/IPT1) were used as an antigen and fixed on slides (Kartell) in an acetone bath. IFAT slides were incubated for 30 min at 37 °C, and negative and positive controls were included in each set of samples assayed. Slides were washed three consecutive times in PBS (10 min each) and incubated for 30 min at 37 °C, with fluorescent anticat IgG or antidog igG-FITC antibody (Sigma Aldrich, St. Louis, MO, USA). The slides were washed three times (10 min each) in PBS and the reactivity of the sera was detected with a Leica DM 4000B fluorescence microscope (Leica, Heerbrugg, Switzerland, 40× magnification). IFAT cutoff was set at 1:80 for CanL, and cats were considered positive for feline leishmaniosis (FeL) at the 1:40 dilution.

Conjunctival swabs from both eyes were stored in 20-ml plastic tubes and kept at −20 °C pending DNA extraction for real-time PCR (qPCR). DNA was extracted using PureLink™ Genomic DNA Mini Kit (Thermo Fisher Scientific K182002, Waltham, MA, USA) following the manufacturer’s instructions. The real-time PCR was performed in a QuantStudio 3 (Life Technology, Waltham, MA, USA) and carried as described from Castelli et al. [23]. qPCR was conducted in 20-µL reactions containing 10 µL of SsoAdvanced Universal Probes Supermix (Biorad, Hercules, CA, USA), 0.25 µM QLeish Probe, 0.3 µM of each primer, and 2 µL of extracted DNA at 10 ng/µL. Tenfold serially diluted *L. infantum* parasite DNA corresponding to 1 × 10^6^ to one parasite per mL was used as standard curve. The thermal cycle was set as follows: initial denaturation for 10 min at 95 ˚C, 40 cycles of denaturation at 95 ˚C for 15 s, and annealing polymerization at 60 ˚C for 35 s [24].

Morbidity and cumulative incidence were recorded. Incidence density rate (IDR) was calculated from the total time (in months) spent by each dog in the study, using the equation: IDR = (Number of positive test / Time each dog observed in months, totaled for all dogs) × 100.

Analyses were performed using the statistical software MedCalc.

## Results

### Canine seroprevalence

Dogs (154) included in the seroprevalence assessment averaged 5.5 years; there were 89 males (57.8%) and 65 females (42.2%); 14 (10.4%) lived exclusively indoors, 60 (44.8%) lived outdoors, and 60 (44.8%) had access to both indoor and outdoor environments.

Owners reported that 89 dogs (57.7%) previously received an antiparasitic treatments, mainly during the spring or summer. On physical examination, 23 dogs (14.9%) showed mild potential CanL clinical signs, including lymph node enlargement and cutaneous lesions, and 19 (12.3%) tested positive on IFAT. No dog tested positive for any other common canine vector-borne disease (Snap^®^ 4Dx Plus, IDEXX).

### Feline seroprevalence

The FeL seroprevalence was 32.9% (27/82), and 15 (18.3%) had an IFAT > 1:40.

### Follow-up phase results

Of the 62 dogs completing the 2-year study, only two developed a positive result to the rapid test and both showed clinical signs on physical examination (lymph nodes enlargement and weight loss) and were positive on IFAT, leading to an estimated CanL morbidity of 3.22% (95% confidence interval (CI) 0.00–7.22).

IFAT testing of saved serum samples found a further nine positive dogs that were negative on the rapid test. These dogs were asymptomatic with no detected clinicopathological changes throughout the study period, and over the study period, their IFAT titers tended to decline following day 180 and several became negative (Table 1). The estimated cumulative test-positive incidence was 14.5% (standard error (SE) 8.77; 95% CI 5.7–23.3), while the incidence density, calculated as the number of new cases / total time at risk (months), was 0.61%

**Table 1:** IFAT titers in rapid test-negative low-positive dogs during follow-up

Dog ID	Day 0	Day 180	Day 360	Day 540	Day 720
9	1:40	< 1:40	1:160	< 1:40	1:80
11	< 1:40	1:80	< 1:40	< 1:40	< 1:40
12	< 1:40	1:80	< 1:40	< 1:40	< 1:40
14	1:80	< 1:40	1:160	1:160	1:80
15	< 1:40	1:160	1:80	< 1:40	< 1:40
35	< 1:40	1:80	< 1:40	< 1:40	< 1:40
37	< 1:40	1:80	< 1:40	< 1:40	< 1:40
45	1:80	< 1:40	< 1:40	NA	< 1:40
53	< 1:40	1:160	< 1:40	NA	< 1:40

*IFAT*, indirect fluorescent antibody test; *NA*, not available

## Discussion

This study found that concurrent dog vaccination and administration of a protective collar is a safe approach to reducing the risk of CanL in dogs living in an endemic environment, although protection achieved was not 100%. CanL control is a key public health objective and represents an important One Health goal, as the disease is highly endemic in canine populations and infected dogs are the primary domestic reservoir of *Leishmania infantum*.

The geographically confined Favignana Island environment provided a valuable setting where hosts, vectors, and pathogens are interacting and there is an opportunity to assess intervention outcomes.

This is the first study to document CanL seroprevalence on Favignana Island, with 12.3% positive dogs and 32.9% positive cats. Higher prevalences are reported for other areas of Sicily, where regional data from the National Reference Centre for Leishmaniasis (CRENAL) indicate prevalence of 20%–40% (unpublished data). The differences likely do not reflect a reduced transmission risk on Favignana but is more typical of a study population consisting exclusively of well-cared-for owned dogs that may have been treated with ectoparasiticides and monitored during previous sandfly transmission seasons. CRENAL data are derived primarily from samples submitted from clinically suspected dogs, and this may lead to an overestimation of prevalence. Retrospective test results in Sicily between 2013 and 2021 reported a 34% canine seroprevalence, confirming the high regional prevalence of CanL [25]. The median CanL prevalence across Italy was estimated at 17.7% [6], and across other Mediterranean basin countries prevalence rates are 10% to > 30%, depending on geographical area and dog population characteristics [10, 26]. These data confirm that Favignana Island represents a high-transmission endemic environment consistent with the broader epidemiological scenario of southern Europe.

The high prevalence of test-positive cats in this study is consistent with prior work that demonstrated that cats act as secondary hosts of *L. infantum* and may represent an alternative infection reservoirs [27, 28] and cats living in endemic areas experience exposure risks comparable to dogs, although feline infection is more frequently subclinical or asymptomatic [25]. The 32.9% feline seroprevalence observed on Favignana exceeds reports from other Italian regions (approximately 11%–12%) [20, 21, 25] and in the Mediterranean basin [27]. It is possible that cats may represent an increased transmission risk in confined insular ecosystems, and preventive strategies for feline populations could be important for control in these settings.

Some of the dogs receiving concurrent vaccination and a protective collar developed IFAT-positive results over the 2-year follow-up period, and two dogs were removed from the study following onset of clinical signs. These results are encouraging and consistent with a reduced occurrence of clinical disease in vaccinated and collar treated dogs. Clinical CanL developed in 8 of 275 vaccinated dogs (4.7%) over two transmission seasons in a prior field trial [11], while in the present study, 3.2% of dogs developed clinical disease, recognizing there are differences in study design and epidemiological context.

There were nine clinically healthy dogs identified as IFAT positive during the study period, and antibody titers declined or reverted to negative in most dogs. These findings may be consistent with immune-mediated control of infection in vaccinated dogs. Vaccination may help to prevent onset of clinical disease and also modulate subclinical infections, and this is consistent with the declining titers observed in this study. This vaccination benefit could be particularly helpful in endemic areas because many infected dogs are asymptomatic at the time of vaccination [29]. Studies have shown that LETIFEND^®^ vaccination reduced circulating immune complex levels in experimentally *L. infantum*-infected dogs compared with unvaccinated controls [30]. Clinical severity is strongly influenced by host immunogenetics and Th1/Th2/regulatory response balance. Circulating immune complexes associated with high antibody titer correlate with disease progression [31], and an uncontrolled humoral response can contribute to disease progression in asymptomatic infected dogs. This study provides a further indication that LETIFEND^®^ vaccination contributes to low and potentially negative IFAT titers, and this may be an important parameter in ameliorating disease progression.

Only two dogs in the trial developed overt disease requiring treatment with a calculated clinical morbidity of 3.22%. The number of affected dogs is epidemiologically relevant, as dogs with clinical disease are more infectious to the sandfly vectors [32–35] and contribute to transmission. Concurrent LETIFEND^®^ vaccination and SCALIBOR^®^ collar administration can reduce both the number of clinically diseased animals and the burden of asymptomatic carriers. This reduction contributes to reduce infection pressure within canine populations and, indirectly, the zoonotic risk to people living in the area. The number of infective sandfly bites does not correlate with subsequent clinical severity in infected dogs, although repeated infective bites may increase the infection risk and could lead to a higher downstream parasite burdens [36].

This concurrent vaccination and protective collar protocol was well tolerated by all dog in the study, with no adverse reactions. Some dogs in the study were retrospectively identified as seropositive at the time of repeat vaccination, and these dogs also did not have any reported adverse reactions at the time of repeat vaccination. This is consistent with safety data for LETIFEND^®^ vaccination in healthy infected dogs and is relevant for field application in endemic areas, where vaccinated dogs are commonly pre-exposed to *L. infantum*. LETIFEND^®^ vaccination is safe in healthy infected animals (Letifend RCP) and can be used in endemic areas. [37].

The study is limited by absence of an untreated control group, and ethical considerations preclude withholding known effective preventive measures in a high-risk endemic area.

## Conclusions

Dogs and cats on Favignana Island are frequently naturally exposed to *Leishmania infantum*. Dogs that received concurrent LETIFEND^®^ vaccination and SCALIBOR^®^ collar application had a low risk of developing clinical leishmaniosis, and most seroconversions were transient and not associated with clinical signs. No adverse events were reported in treated dogs. Concurrent vaccination and topical vector control helps to limit clinical CanL development and reduce the subclinical infection burden in dogs in this high-risk endemic areas. Integrated preventive strategies and regular monitoring represents a valuable approach for improving dog health and reducing zoonotic risks.

## Data Availability

Data supporting the main conclusions of this study are included in the manuscript.
